# Genomic profiling distinguishes familial multiple and sporadic multiple meningiomas

**DOI:** 10.1186/1755-8794-2-42

**Published:** 2009-07-09

**Authors:** Yiping Shen, Fabio Nunes, Anat Stemmer-Rachamimov, Marianne James, Gayatry Mohapatra, Scott Plotkin, Rebecca A Betensky, David A Engler, Jennifer Roy, Vijaya Ramesh, James F Gusella

**Affiliations:** 1 Molecular Neurogenetics Unit, Center for Human Genetic Research, Massachusetts General Hospital, Boston, MA, 02114, USA; 2 Department of Neurology, Massachusetts General Hospital, Charlestown, MA, 02129, USA; 3 Molecular Neuro-Oncology and Pathology Laboratories and Cancer Center, Massachusetts General Hospital and Harvard Medical School, Boston, MA, 02114, USA; 4 NF Clinic, Pappas Center for Neuro Oncology, Massachusetts General Hospital and Harvard Medical School, Boston, MA, 02114, USA; 5 Department of Biostatistics, Harvard School of Public Health, Boston, MA, 02115, USA

## Abstract

**Background:**

Meningiomas may occur either as familial tumors in two distinct disorders, familial multiple meningioma and neurofibromatosis 2 (NF2), or sporadically, as either single or multiple tumors in individuals with no family history. Meningiomas in NF2 and approximately 60% of sporadic meningiomas involve inactivation of the *NF2 *locus, encoding the tumor suppressor merlin on chromosome 22q. This study was undertaken to establish whether genomic profiling could distinguish familial multiple meningiomas from sporadic solitary and sporadic multiple meningiomas.

**Methods:**

We compared 73 meningiomas presenting as sporadic solitary (64), sporadic multiple (5) and familial multiple (4) tumors using genomic profiling by array comparative genomic hybridization (array CGH).

**Results:**

Sporadic solitary meningiomas revealed genomic rearrangements consistent with at least two mechanisms of tumor initiation, as unsupervised cluster analysis readily distinguished tumors with chromosome 22 deletion (associated with loss of the *NF2 *tumor suppressor) from those without chromosome 22 deletion. Whereas sporadic meningiomas without chromosome 22 loss exhibited fewer chromosomal imbalance events overall, tumors with chromosome 22 deletion further clustered into two major groups that largely, though not perfectly, matched with their benign (WHO Grade I) or advanced (WHO Grades II and III) histological grade, with the latter exhibiting a significantly greater degree of genomic imbalance (P < 0.001). Sporadic multiple meningiomas showed a frequency of genomic imbalance events comparable to the atypical grade solitary tumors. By contrast, familial multiple meningiomas displayed no imbalances, supporting a distinct mechanism for the origin for these tumors.

**Conclusion:**

Genomic profiling can provide an unbiased adjunct to traditional meningioma classification and provides a basis for exploring the different genetic underpinnings of tumor initiation and progression. Most importantly, the striking difference observed between sporadic and familial multiple meningiomas indicates that genomic profiling can provide valuable information for differential diagnosis of subjects with multiple meningiomas and for considering the risk for tumor occurrence in their family members.

## Background

Meningiomas, which arise from arachnoidal cap cells of the leptomeninges, display an annual incidence 5.5 per 100,000, accounting for ~20% of all primary intracranial tumors [1,2]. They may be classified histologically into three grades, according to World Health Organization (WHO) criteria [3]: WHO grade I meningiomas (~90%) are slow growing benign tumors; WHO grade II meningiomas (6–8%) are described as atypical and display increased cellularity and mitotic activity; and WHO grade III meningiomas (2–3%) are termed anaplastic or malignant and have a high recurrence risk. Meningiomas commonly occur as sporadic solitary tumors in the general population and may be found at autopsy in asymptomatic individuals. They also occur in ~50% of individuals with the inherited disorder neurofibromatosis 2 (NF2) [4], which involves inactivation of the *NF2 *gene on chromosome 22, encoding the merlin tumor suppressor [5,6]. About 60% of sporadic solitary meningiomas occur due to merlin inactivation and usually display loss of one copy of chromosome 22, while the mechanism of tumorigenesis in the remaining 40% remains unknown [3,7,8]. In 1–8% of patients, meningiomas present as multiple tumors either due to a predisposing *NF2 *mutation or due to noncontiguous spread of a single sporadic tumor [9-15]. Non-NF2 multiple meningiomas may appear either as sporadic or familial cases [9,11,12,16-20]. Like sporadic solitary meningiomas, sporadic multiple meningiomas may display somatic *NF2 *mutations [14,16], whereas familial multiple meningiomas do not [16]. Like NF2, familial multiple meningioma has been reported to show autosomal dominant inheritance [21-23], but it does not show linkage to the *NF2 *locus [24] and tumors from at least one kindred exhibit immunoreactivity for merlin [18]. Together, the accumulated evidence indicates that in addition to inactivation of merlin, there are one or more other mechanisms for initiating formation of a meningioma.

Array Comparative Genomic Hybridization (array CGH) is a powerful whole genome profiling technology capable of detecting both DNA copy gain and loss that has the potential for unbiased classification of tumor specimens based upon the constellation of genomic alterations that they possess. Here we have applied this technology to examine genomic imbalance events in familial multiple meningioma, in comparison with a cohort of sporadic solitary meningiomas and sporadic multiple meningiomas. Our findings indicate that while genomic profiling can implicate an etiology for sporadic tumors and suggest candidates for initiation or progression loci, the revelation of an absence of gross genomic imbalance events in familial multiple meningioma suggests that it is a distinct genetic entity.

## Methods

### Tumor specimens

Patients and tumors were ascertained through the Neurofibromatosis Clinic at Massachusetts General Hospital as previously reported [13,16]. Excess tumor tissue not required for diagnosis was flash frozen in liquid nitrogen. Genomic DNA was extracted from tumor tissue as previously described [16]. When available, pathology sections were re-reviewed using 2007 WHO criteria [3]. Pathology sections were not available for the 5 sporadic multiple and 4 familial multiple meningiomas. In addition, some of the sporadic solitary meningiomas could not be scored for histological subtype, particularly tumors that were more anaplastic (in the grade II and III tumors). Specimens that are "not scorable" had small fragments of tissue, insufficient for determination of histological subtype. In meningiomas WHO grade II or III, disruption of the tumor's growth pattern by hypercellular foci and sheeting often does not allow the accurate classification of the underlying histological subtype. Specific patterns associated with WHO Grade II or III meningiomas (such as rhabdoid, papillary etc.) have been specified, as advised by the WHO classification.

The characteristics of the sporadic solitary tumors are shown in Table 1. This tumor set partially overlaps with the set investigated previously by Nunes et al. [25] and contains eight tumors reported in that study to show deletion at the *NF2 *and *EBP41L3 *(DAL1) genes. Of the 9 multiple meningiomas studied here, 3 (2 sporadic and 1 familial) were investigated previously in Heinrich et al. [16] for deletion of the *NF2 *and *EBP41L3 *(DAL1) genes. This study was approved by the Institutional Review Board of Massachusetts General Hospital and informed consent was obtained from all study subjects.

**Table 1:** Characteristics of Sporadic Solitary Meningiomas

Tumor Designator	Patient Age	Gender	Tumor Grade	Histology	Recurrence
7B	79	F	I	Fibroblastic	no
8B	33	F	I	Fibroblastic	no
9B	50	F	I	Fibroblastic	no
11B	69	F	I	Fibroblastic	no
12B	45	F	I	Fibroblastic	no
13B	43	F	I	NS	no
14B	59	F	I	Fibroblastic	n/a
15B	55	F	I	Fibroblastic	no
46B	36	F	I	NS	no
48B	41	M	I	Papillary	n/a
50B	63	M	I	NS	no
51B	80	M	I	Transitional	no
52B	39	F	I	NS	no
55B	72	F	I	Fibroblastic	no
56B	70	F	I	Transitional	no
57B	69	F	I	NS	no
58B	39	F	I	Microcystic	no
61B	n/a	F	I	Meningothelial	no
63B	52	M	I	Transitional	no
65B	58	F	I	Meningothelial	no
66B	59	F	I	NS	no
68B	35	F	I	NS	no
71B	34	F	I	Meningothelial	yes
73B	77	F	I	Fibroblastic	no
75B	67	F	I	NS	no
10A	81	F	II	Transitional	yes
17A	58	M	II	Fibroblastic	no
20A	75	F	II	NS	no
26A	77	F	II	Meningothelial	no
27A	59	F	II	NS	no
29A	46	M	II	Transitional	no
30A	59	M	II	Transitional	no
34A	54	M	II	NS	no
35A	58	M	II	Fibroblastic	no
37A	58	F	II	NS	no
38A	78	M	II	Meningothelial	yes
39A	85	M	II	Fibroblastic	yes
40A	36	M	II	NS	yes
42A	69	F	II	Fibroblastic	no
43A	35	M	II	Transitional	no
44A	50	F	II	Transitional	no
45A	56	M	II	NS	no
47A	53	F	II	NS	no
49A	74	F	II	Transitional	no
54A	78	M	II	Transitional	no
67A	54	F	II	NS	no
72A	41	F	II	Meningothelial	no
76A	63	M	II	Meningothelial	no
77A	29	F	II	Meningothelial	yes
80A	59	F	II	NS	no
81A	62	F	II	Angiomatous	no
83A	60	M	II	Meningothelial	no
1M	52	F	III	NS	n/a
2M	73	F	III	NS	yes
3M	46	M	III	NS	no
4M	73	F	III	NS	yes
21M	57	F	III	Fibroblastic	yes
22M	60	F	III	Meningothelial	no
33M	55	M	III	NS	no
126M	70	M	III	NS	no
127M	44	F	III	NS	yes
128M	28	F	III	NS	yes
129M	62	M	III	NS	yes
130M	80	M	III	NS	no

NS = not scorable (see Methods); n/a = data not available

### MLPA analysis

For *NF2 *gene dosage analysis of all multiple meningiomas and a subset of ten sporadic solitary meningiomas, we performed multiplex ligation-dependent probe amplification (MLPA) using kit P044-NF2 following the manufacturer's protocol (MRC-Holland, Amsterdam, The Netherlands; ). Briefly, 200 ng of DNA were incubated overnight with probe mix (P044) at 60°C. A 15-min ligation was performed afterwards at 54°C. Ten μl of ligation products were used for PCR using FAM-labeled universal primers. PCR products were resolved using ABI 3730XL DNA analyzer and peaks were quantified by Genotyper software. The data were analyzed and plotted by a custom excel based program.

### Array CGH

Array CGH was performed for all tumors using Agilent cDNA microarrays as previously described [25]. These arrays contain 14,160 cDNA clones (Agilent Technologies, Palo Alto, CA) with a median interval between mapped elements of 100 kb, with 92.8% of intervals <1 Mb and 98.6% <3 Mb [26]. Briefly, one μg of tumor or normal genomic DNA (male or female) was digested with *Dpn*II (New England Biolabs, Beverly, MA, USA), purified with DNA Clean & Concentrator (Zymo Research, Orange, CA, USA) and labeled with Cy3-dCTP or Cy5-dCTP (Amersham Bioscience, UK) using the Bioprime DNA Labeling System (Invitrogen Life Technologies, Carlsbad, CA, USA). Labeled DNA was precipitated using isopropanol together with Cot-1 DNA (Invitrogen Life Technologies, Carlsbad, CA, USA), which was used to block repetitive sequences. The probe pellet was then washed with 70% ethanol, dried and dissolved in hybridization buffer consisting of 50% formamide, 2 × SSC, 10% dextran sulphate, 2% SDS and 10 μg/μl yeast tRNA. Dye swap experiments were performed in all cases. One common male control DNA and one common female control DNA were used for all hybridizations. The comparison between test sample and control sample was done in a sex mismatched manner for most cases. Hybridizations were performed in sealed chambers for 60 h at 37°C. After hybridization, slides were rinsed in 2 × SSC, 0.5% SDS at room temperature; washed for 20 min in 2 × SSC, 50% formamide at 50°C; 20 min in 2 × SSC, 0.1% NP-40 at 50°C; and 10 min in 0.2 × SSC at 50°C. Slides were then air dried by centrifugation before imaging. Sixteen-bit TIF images were collected using an Axon 4000B scanner and processed initially using GenePix Pro 4.1 (Axon Instruments, Inc. Union City, CA). Defective spots were flagged by visual inspection of the images and subsequently custom software was used to exclude spots that demonstrated low signal to noise ratios [27]. The baseline CGH level (two genomic copies) was calculated by the software as the median Cy3 and Cy5 ratio of all clones analyzed. Dye swap experiments were merged to calculate the mean and standard deviation of the signal for each target clone. The normalized values were transformed to log2 format to ensure equal weighting for gains and losses.

For the familial multiple meningiomas, array CGH was also performed using the Agilent 44 K CGH array (Agilent Technologies, Palo Alto CA), which contains more than 43,000 coding and noncoding human oligonucleotide sequences with an overall median probe spatial resolution of 43 kb (24 kb within RefSeq genes) [28]. Oligo array CGH was performed according to the manufacturer's protocol. Briefly, three μg of DNA (both test sample and control sample) were double digested with Alu I and Rsa I and subsequently purified with QIAprep Spin Miniprep kit (QIAGEN GmbH, Hilden, Germany). Digested samples were labeled with Cy3-dUTP or Cy5-dUTP (Amersham Bioscience, UK) using the Bioprime array CGH DNA Labeling System (Invitrogen Life Technologies, Carlsbad, CA, USA); paired samples were mixed and subsequently purified by MicroCon YM-30 (Millipore, Billerica, MA, USA). Labeled probes were mixed with Cot-1 DNA (Invitrogen Life Technologies, Carlsbad, CA, USA), blocking solution and hybridization solution (Agilent Technologies, Palo Alto, CA, USA) and hybridized to a 44 K CGH array. Hybridizations were performed in a 65°C oven on rotating rack for 40 hrs. Arrays were washed with wash 1 and 2 solutions (Agilent Technologies, Palo Alto, CA, USA) and scanned immediately using the Agilent scanner. Images were analyzed using Feature Extraction software and data visualized with CGH analytics (Agilent Technologies, Palo Alto, CA, USA).

### Data analysis

Imbalanced chromosome segments (ICS) were identified by change-point analysis using a circular binary segmentation algorithm [29]. An ICS is defined in this study as a segment identified by change-point analysis with mean log2 ratio >0.20 (gain) or <-0.20 (loss) based upon a separation of 2 standard deviations from the mean. The binary segmentation procedure applies the test recursively until no more changes are detected in any of the segments obtained from the change-points already found. When no change point was found over the whole chromosome or chromosome arm and the mean log2 value of the whole chromosome or chromosome arm was either >0.20 or <-0.20, the whole chromosome or chromosome arm was considered imbalanced. The calling of whole chromosome or chromosome arm imbalance was therefore dependent upon the even distribution (no change point) and mean of all data points involved in the whole chromosome or chromosome arm, rather than on how many probes have log2 ratio above a pre-defined number. The physical map positions of subchromosomal segments were based on NCBI build 33. The Kolmogorov-Smirnov (KS) test was used for comparing the ICS number difference between tumor groups. Hierarchical cluster analysis was performed using the CGHsmooth [30] transformed whole genome dataset. Probes with no copy change are designated as 0, probes with one copy loss designated as -1, probes with a copy number gain designated as 1, and probes with amplification designated as 2. Uncentered correlation using the means of all pair-wise data points for distance metrics (average linkage) was used for cluster analysis [31].

## Results

### Array CGH analysis of sporadic solitary meningiomas

**Figure 1:**
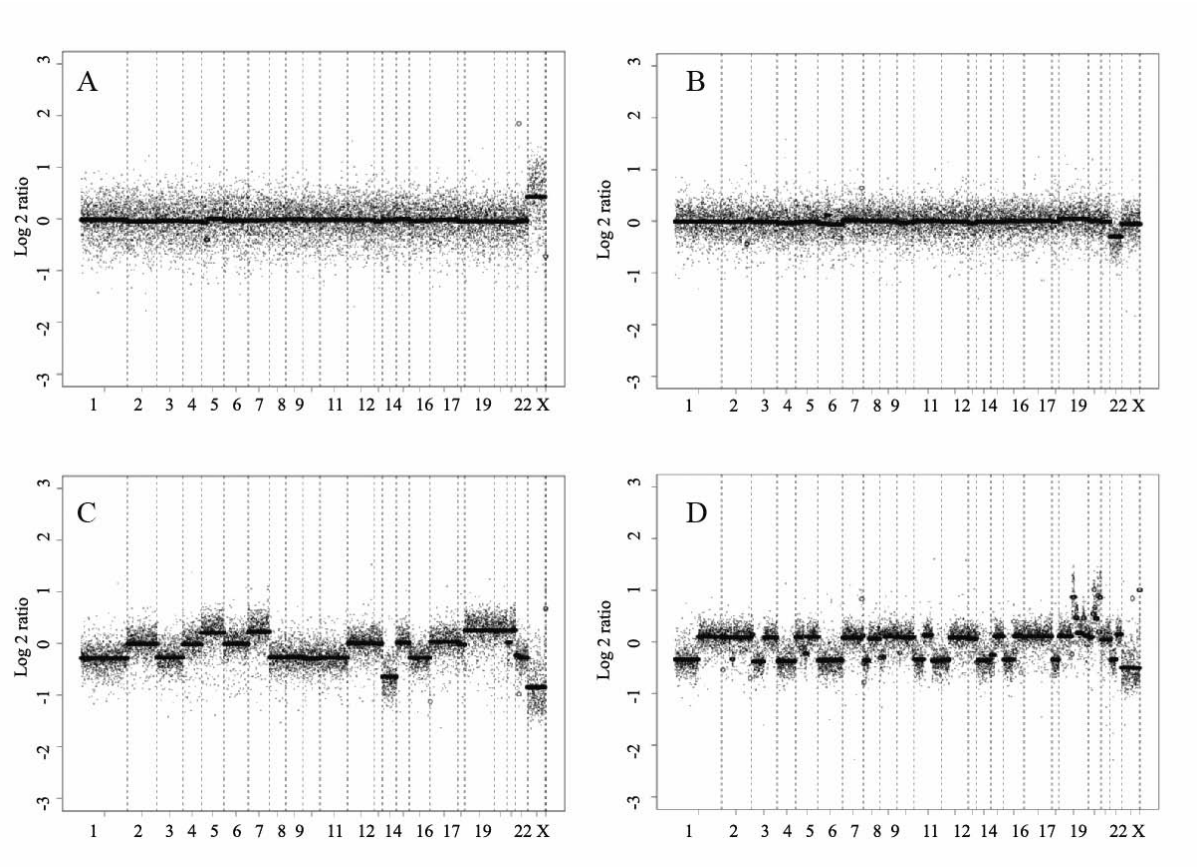
**Representative array CGH profiles of meningiomas**. Segmentation analysis of array CGH results generated from Agilent cDNA arrays using four representative sporadic solitary meningiomas is shown: A: Benign meningioma (68B) without imbalanced chromosome segments (ICS). The X chromosome imbalance is due to use of a sex-mismatched standard DNA as a positive control; B: Meningioma with chromosome 22 deletion as the only imbalance event (42A); C: Atypical meningioma with both gain and loss ICS (43A); D: Malignant meningioma with many ICS and focal amplification (129M). X-axis: clones are ordered from chromosome 1 to 22, X and Y and within each chromosome, clones are arranged following their physical map order from short arm telomere to long arm telomere. Y-axis: log2 ratio (test/control) of array CGH signal for each individual probe (scattered dots) and for segments of consistent dosage (solid lines) defined by binary segmentation. The baseline (no copy number change) is 0; segments above the baseline indicate gain of copy number and segments below the baseline indicate loss of copy number.

**Figure 2:**
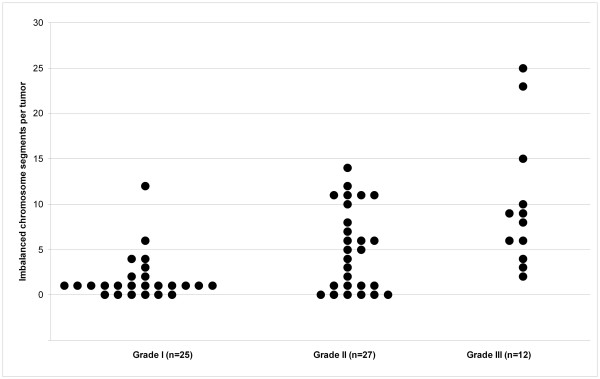
**The distribution of Imbalanced Chromosome Segments in three groups of sporadic solitary meningiomas**. Each dot represents an individual tumor. Y-axis indicates the total number of imbalanced chromosome segments (ICS) in each tumor; X-axis shows three groups of meningioma of different grades. The total number of ICS ranges from 0–12 in benign meningiomas, 0–14 in atypical meningiomas and 2–25 in malignant meningiomas.

To provide a basis for comparison of tumors from subjects with multiple meningiomas, we first used two-color array CGH with Agilent human cDNA chips to generate genomic profiles for 64 sporadic solitary meningiomas: 25 WHO grade I (benign), 27 WHO grade II (atypical) and 12 WHO grade III (malignant). Representative array CGH profiles with segmentation analysis are shown in Figure 1 (two benign meningiomas: Panels A and B; one atypical: Panel C; one malignant meningioma: Panel D). We observed a progressively increasing number of ICS in higher grade tumors (see Figure 2 for the distribution of ICS in each tumor group). The difference in total ICS (P < 0.001), as well as in gain events (P = 0.01) or loss events (P < 0.001) considered separately, was significant between benign and high grade tumor groups (atypical and malignant) whereas the difference in total ICS between atypical and malignant tumor groups was not (P = 0.47).

**Figure 3:**
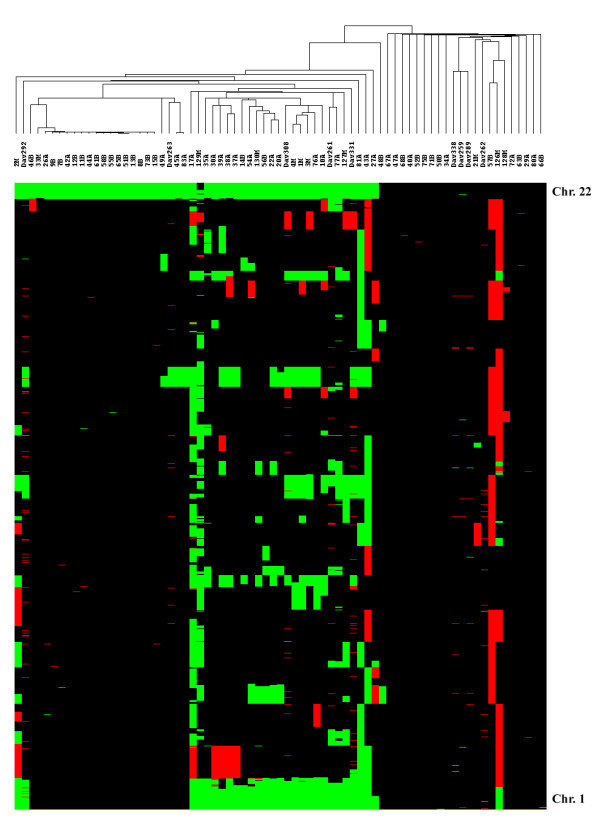
**Tree view of unsupervised hierarchical cluster analysis of meningiomas based upon array CGH data**. A cluster diagram generated using array CGH data from Agilent cDNA array analysis of 63 sporadic solitary meningiomas, 5 sporadic multiple meningiomas and 4 familial multiple meningiomas is shown above a HEAT map showing the occurrence of imbalanced chromosome segments (ICS) (green = loss; red = gain as defined in Methods) relative to control DNA observed across the genome from chromosome 22 (top) through chromosome 1 (bottom). The dataset segregated into two branches based upon chromosome 22 deletion status, with tumors deleted for chromosome 22 on the left and tumors without chromosome 22 deletion on the right. Of the sporadic multiple meningiomas, DAV331, DAV308 and DAV292 are from the same person (but do not show identical ICS patterns) and DAV261 and DAV263 are each from different subjects; of the familial multiple meningiomas, DAV259 and DAV262 are each from different individuals in two different families while DAV289 and DAV338 are from the same subject, representing a third family.

The above analysis revealed a number of recurrent ICS, shown in the HEAT map of Figure 3, that are largely consistent with previous studies using a variety of technologies. Deletion events occurred frequently on chromosomes 22 (71%), 1 (39%), 14 (25%), 6 (22%), 18 (20%), 3 (17%), 10 (16%), 9 (13%), 4 (13%), 11 (11%) and X (11%). Gain events occurred on chromosome 1, 17 and 20 (11%, 12.5% and 11% respectively). The predominant event in benign meningiomas was 22q deletion, in 64% of the tumors. In atypical meningiomas, 22q deletion occurred in 67% of the tumors, followed in frequency by deletion of 1p (52%), 14q (41%) and 6q (26%), whereas in malignant meningiomas, 1p deletion was equally as frequent as 22q deletion, occurring in 75% of these tumors, followed by deletion of 6q, 9p and 10q (all at 50%), 3p at 42%, and gain of 20q (33%). These data are consistent with the accepted view that deletion of chromosome 22 is an early event responsible for initiation of the major fraction of sporadic meningiomas, but that a significant minority of tumors arises via some other mechanism. It is also thought that meningiomas progress via a stepwise pathway, i.e., atypical and malignant meningiomas develop due to accumulation of additional genomic lesions [32]. Our whole genome profiling data for sporadic solitary meningiomas largely support such a model, as there was significantly (P < 0.001) greater genomic instability in high grade tumors than in low grade tumors.

### Array CGH analysis of multiple meningiomas

**Figure 4:**
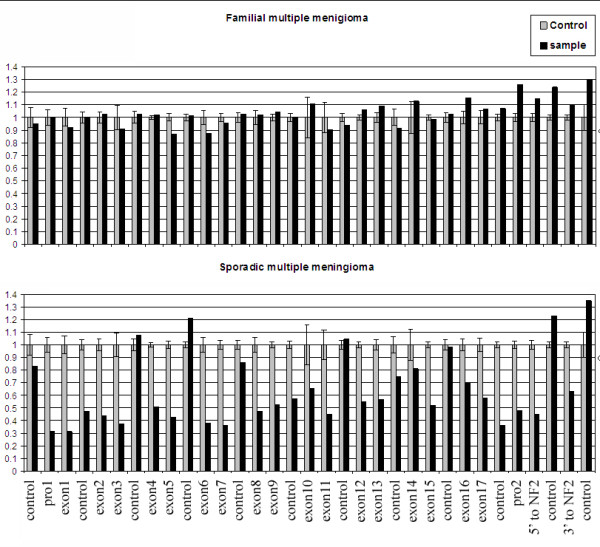
**MLPA analysis of sporadic and familial multiple meningiomas**. Representative MLPA analyses of a familial multiple meningioma (DAV338, top panel) and a sporadic multiple meningioma (DAV308, bottom panel) are shown with probe IDs on the X-axis. There are 21 MLPA probes for the *NF2 *gene (one probe for each exon, two probes for the promotor region and one probe on either side of the gene) and 12 control probes located on other chromosomes. The Y-axis shows the relative probe peak height as an indication of copy number. The relative peak heights were calculated by normalizing the peak height of control probes between normal control DNA and tumor DNA. Gray bars indicate the mean peak height with standard deviation from 10 normal control DNAs. Black bars indicate the peak height for the tested tumor DNA. The top panel reveals no deletion of the *NF2 *gene while the bottom panel is consistent with deletion of one copy of the *NF2 *gene, including its flanking regions. All multiple meningiomas and a sampling of 10 sporadic solitary meningiomas were tested by MLPA, with results at this locus identical to those from array CGH.

Co-occurrence of multiple meningiomas in individuals who do not have germline *NF2 *mutation occurs far less frequently than do solitary meningiomas. We obtained four familial multiple meningiomas from three unrelated individuals (all female), along with five tumors from three individuals with sporadic multiple meningiomas with DNA quality adequate for this study. Previous mutational analysis identified somatic *NF2 *mutations in the sporadic multiple meningiomas but not in the familial cases [16]. We performed gene dosage analysis by MLPA to determine whether there was somatic loss of *NF2 *in these tumors (Figure 4). One copy of *NF2 *was deleted in all sporadic multiple meningiomas, consistent with the known "two-hit" mechanism of tumorigenesis involving the merlin tumor suppressor. However, none of the familial multiple meningiomas showed a loss at *NF2*. These findings, and the prior mutation analyses, support the notion that familial multiple meningiomas do not arise by inactivation of the *NF2 *gene.

By genomic profiling using two-color array CGH with Agilent human cDNA chips, we found that the sporadic multiple meningiomas had several recurrent ICS, including deletion of the whole chromosome 22 in all 5 tumors, deletion of 1p, all of chromosome 14, and part or all of chromosome 10 in 4 of 5 tumors and gain of the whole chromosome 20 in 2 of 5 tumors. This pattern was similar to that seen in Grade II sporadic solitary meningiomas, as was the average ICS/tumor of 5.8 ± 2.7 in sporadic multiple meningiomas and 5.0 ± 4.5 in atypical sporadic solitary meningiomas. In stark contrast, familial multiple meningiomas did not show any ICS.

### Cluster analysis of meningioma array CGH data

To provide an unbiased global view of these genomic profiles, we performed unsupervised hierarchical cluster analysis using the CGHsmooth transformed dataset [30]. The cluster analysis was done by average linkage using the means of all pair-wise data points as distance metrics, yielding the tree presentation shown in Figure 3. The unsupervised cluster analysis split the whole tumor set into two main branches, one comprised of tumors with chromosome 22 deletion (left side of Figure 3) and one comprised of tumors without chromosome 22 deletion (right side). The procedure further subdivided the sporadic solitary meningiomas with chromosome 22 deletion into two major groups, with the exception of four outliers (27A, 2M, 43A and 81A). One group contained 14 Grade I tumors along with 7 higher Grade tumors (6 II and 1 III). Notably, 13 of these 14 Grade I meningiomas were grouped into a single subcluster characterized by loss of chromosome 22 as the only major imbalance event. The second major group was comprised mainly of meningiomas of advanced grade (11 Grade II and 7 Grade III out of 20 solitary sporadic tumors), although 2 tumors classified as Grade I were also present. These tumors had more ICS than those in the first group and implicate progression genes at loci involving losses on chromosomes 1p, 14q, 6q, 18, 9p and 10q and gains on chromosomes 17q and 20.

Though the cluster analysis generally reflected, with some exceptions, the benign or advanced histological grading of those solitary sporadic meningiomas showing chromosome 22 loss, the solitary sporadic meningiomas without chromosome 22 loss were not at all grouped according to their grade. There were very few imbalance events for most benign and atypical meningiomas of this type. Indeed, there was a notable subgroup of high grade tumors in both chromosome 22-defined groupings (such as 21M, 33M, 128M, 26A, 29A, 34A, 40A, 42A, 44A, 45A, 72A, 80A, 83A) that did not exhibit extensive genomic imbalance (ICS = 4). Many of these tumors exhibited no chromosome 22 deletion. Overall, we observed a significant difference in the frequency of non-chromosome 22 genomic imbalance events between solitary sporadic tumors with chromosome 22 deletion and those without (P = 0.006). Though the difference was confined to tumors of advanced grade, it may have contributed, in addition to chromosome 22 loss, to the segregation of the solitary sporadic meningiomas into two main branches (Figure 3). In any event, while our data are generally consistent with a traditional multistep model of tumor progression for those meningiomas with chromosome 22 deletion, where *NF2 *inactivation is likely the initiating event, this may not apply to tumors without chromosome 22 deletion, which apparently harbor unidentified genetic lesions that may result in more direct progression without the accumulation of multiple ICS.

In the unsupervised cluster analysis, familial multiple meningiomas were cleanly distinguished from the sporadic multiple meningiomas. The latter were spread across the left hand side of the cluster diagram in Figure 3, among the tumors with chromosome 22 loss, one with the outliers mentioned above, one amid several atypical tumors in the grouping of mainly benign tumors and three in the grouping mainly of atypical and malignant tumors. By contrast, the familial multiple meningiomas clustered among the solitary sporadic tumors to the right of Figure 3, characterized by the absence of chromosome 22 loss.

To determine whether the familial multiple meningiomas might harbor smaller imbalance events that were missed by the relatively low resolution cDNA chip, we repeated the array CGH analysis of these particular familial multiple meningioma samples using a newer, higher resolution Agilent human oligo array. Even with this higher resolution array, the familial multiple meningioma tumors showed no ICS in any of the four tumors, supporting the view that familial multiple meningiomas are fundamentally different from sporadic multiple meningiomas both in terms of *NF2 *mutation and genomic profile.

## Discussion

We demonstrate here that histologically similar tumors with a proposed different genetic etiology can be differentiated based upon their genomic profiles using array CGH as an effective method to generate informative profiles in a genome-wide manner. Previously, large-scale gene expression profiling using RNA extracted from tumor tissue has demonstrated its utility in classifying different tumor subtypes of leukemia and lymphoma [33,34] and solid tumors [35,36]. It has also been applied to study the molecular signatures of meningiomas of different grades and locations [37-41]. Notably, in a recent study, gene expression patterns were shown to be predictive of major cytogenetic patterns seen in meningiomas (chromosome 22 or 22q loss, loss of 1p36 alone, complex karyotypes with loss of 1p36 and/or 14q and diploid) based upon altered expression of a combination of genes within the regions of altered dosage and genes outside these regions [42]. Each individual gene expression change represented an apparent correlate of the corresponding cytogenetic abnormality that could potentially play a role in meningioma initiation or progression. In particular, those altered genes not actually located in a region of cytogenetic abnormality may well represent components of pathways critical to meningioma formation. While it is difficult to assign such a role to each individual gene without additional studies, the overall pattern observed was clearly associated with patient outcome, though not with tumor histopathology.

Like gene expression profiling, whole genome profiling by array CGH using DNA extracted from tumor tissue has also shown its power to differentiate solid tumors of both mouse and human [27,43-45]. Solid tumors often exhibit genomic imbalance events and some of these imbalance events are specific to certain types or subtypes of tumors. In view of the stable nature of DNA compared with RNA, array CGH is a potentially more widely applicable, easy and effective method for histo-molecular classification. In addition, array CGH provides information about genome stability of the tumor, which may be critical information for assessing its biological state and pathogenic potential.

Our array CGH analysis of sporadic solitary meningiomas detected a wide variety of genomic events, largely consistent with those which have been detected previously by a variety of cytogenetic and molecular techniques, and have often been associated with tumor progression [32,46-52]. Notably, through unsupervised cluster analysis using our entire genome array CGH dataset, we observed a clean separation of meningiomas with or without chromosome 22 deletion for both solitary and multiple meningiomas. Chromosome 22 deletion is a frequent mechanism for somatic inactivation of the *NF2 *gene, thought to account for ~60% of meningiomas [7]. The initiating event(s) in meningiomas that retain chromosome 22 has not yet been identified. The distinction between the two groups of meningiomas by array CGH cluster analysis apparently reflects the existence of at least two main types of genetic mechanism for meningioma tumorigenesis. Since no consistent imbalance event predominates among tumors without chromosome 22 deletion, there may be a greater genetic diversity in the initiation of these tumors. The lower number of ICS in tumors that retain chromosome 22 is consistent with the lack of genomic events detected previously using multi-allele marker techniques [53].

Our data also revealed segregation, based upon a variety of genomic events, of tumors that lost chromosome 22 into two major groups, one consisting predominantly of benign tumors and the other predominantly of tumors of advanced histological grade (Figure 3). The cluster analysis therefore illustrates some potential utility and power of array CGH profiling as an objective means for tumor subclassification and subgrouping. Due to the existence of genetic heterogeneity among meningiomas and the nature of histological examination, we did not expect all tumors to be assigned by cluster analysis precisely in concert with their pathological grade and indeed, that was the case. However, the tendency, with some exceptions, of Grade I tumors to cluster separately from higher grade tumors suggests that further refinement of the genomic profiling strategy is warranted, in combination with more extensive molecular analysis to determine whether differences are due to misclassification by array CGH or rather represent tumors whose molecular changes and predicted behavior are not efficiently recognized in the standard histological examination.

Whole genome array CGH analysis revealed a significant distinction between sporadic meningiomas with or without chromosome 22 deletion. Advanced grade tumors with chromosome 22 deletion had significantly more imbalance events in the rest of the genome than those without, suggesting not only a different genetic etiology, but also a different pathway for tumor progression. This distinction also raises the possibility that prior chromosome 22 loss itself may contribute directly to subsequent genomic instability. Therefore, the array CGH analysis points to the need to unravel potential alternative genetic pathways for a complete understanding of meningioma tumorigenesis. The cDNA array used here did not provide a sufficiently dense coverage to identify small regions of loss that could target specific suppressor genes and we detected no cases of homozygous deletion. There may be potential clues to alternative pathways revealed by the amplification events readily detected in some tumors, though these also typically covered relatively large segments and occurred primarily in malignant meningiomas. For example, meningioma 126M displayed amplification of an ~500 kb region (1.9–2.4 Mb) on chromosome 11 spanning, among other genes, the loci for insulin (*INS*) and insulin-like growth factor 2 (*IGF2*), which has been implicated previously in some meningiomas through expression studies [41]. However, the same tumor also displays larger amplifications on chromosome 1 (78.6–87.5 Mb) and chromosome 17 (41.8–64.0 Mb), both of which contain numerous genes that could potentially play a role in meningioma growth. Similarly, tumor 128M displayed a focal amplification of the c-myc oncogene (*MYC*) on chromosome 8, whose expression may correlate with proliferative index and help predict recurrence of meningiomas [54,55], but the same tumor also had a high level gain on chromosome 17 (49.2–64.0 Mb), overlapping with the large region amplified in 126M. When compared with all tumors that showed a gain on chromosome 17, the minimum shared segment is 58.9–64 Mb and spans more than 40 genes, including such known growth control-related genes as the growth hormone-chorionic somatomammotropin cluster and a protein kinase C family member (*PKCA*) among others. One benign meningioma (63B) had a focal amplification in a gene-rich segment on chromosome 12 (56.19–56.45 Mb), which includes *CDK4*, a known regulator of G1-S progression that phosphorylates the retinoblastoma gene product, Rb, along with several other potential candidates. These disparate events support the possibility that different mechanisms of progression may occur in different tumors but their large size, inconsistent occurrence and lack of a unified predictive pattern suggests that an integration of detailed genomic, genetic, gene expression and functional studies will be needed to delineate the array of alternative pathways to meningioma growth *in vivo*.

Most importantly in our study, array CGH analysis revealed strikingly different genomic profiles between familial multiple meningioma and sporadic multiple meningioma. Though this represents only a small number of tumors and even a single case of loss in one familial multiple meningioma of chromosome 22 or of one of the other chromosomal regions altered in the sporadic multiple meningiomas would reduce the apparent distinction, the absence of all genetic rearrangements in the familial multiple meningiomas is noteworthy. For example, the likelihood that all 4 familial multiple tumors retained chromosome 22 (based upon its overall rate of retention in meningiomas) is ~0.04. Combined with the absence of any other genomic rearrangement in all 4 such tumors, the retention of chromosome 22 provides further evidence that familial multiple meningioma is a genetically distinct tumor predisposition syndrome. Given that *NF2 *mutation was not seen and that chromosome 22 deletion is not a shared feature in these tumors, we believe that one or more other tumor suppressor genes or oncogenes is responsible for the tumor initiation of familial multiple meningioma. Although no genomic imbalance event was identified, even with the higher resolution oligonucleotide array, it is conceivable that loss of heterozygosity without copy number change may be uncovered using high density single nucleotide polymorphisms (SNP) arrays. The finding that sporadic multiple meningiomas harbor a similar level of genomic imbalance events to atypical solitary meningiomas should also draw attention to the potential for greater subarachnoid spreading or higher aggressiveness that would produce a worse prognosis than familial multiple meningiomas. Further, as sporadic multiple meningioma and familial multiple meningioma cannot be distinguished histopathologically, array CGH can provide an important means of differential diagnosis.

## Conclusion

Genome profiling using array CGH can be a valuable complement to histopathology for routine tumor diagnosis and grading and for implicating etiology of meningiomas. Moreover, it can aid differential diagnosis of multiple meningiomas, distinguishing sporadic and familial forms, with potential clinical implications for the risk of meningioma occurrence in other members of the family.

## Competing interests

The authors declare that they have no competing interests.

## Authors' contributions

YS performed the array CGH experiments, data analysis and drafted the manuscript, FN coordinated collection and processing of multiple meningioma samples, AS provided expert neuropathological assessment of tumors, MJ participated in molecular analysis of tumors and sample collection, GM participated in array CGH experiments, SP provided clinical assessment of patients and tumors, RAB and DAE helped analyze the CGH data, created some illustrative plots, and contributed to the methods and results sections of the manuscript, JR banked and processed tumor samples for DNA, VR participated in design of the study and molecular analysis of tumors, and JFG conceived the study and participated in its design and coordination and helped to draft, revise and re-revise the manuscript. All authors read and approved the manuscript.

## Pre-publication history

The pre-publication history for this paper can be accessed here:


